# Development and validation of the Maxillary Canine Aesthetic Index

**DOI:** 10.1002/cre2.133

**Published:** 2018-10-26

**Authors:** Koenraad Grisar, Gilles Claeys, Margot Raes, Emad Ali Albdour, Guy Willems, Constantinus Politis, Reinhilde Jacobs

**Affiliations:** ^1^ OMFS IMPATH Research Group, Department of Imaging and Pathology, Faculty of Medicine University Leuven Belgium; ^2^ Orthodontics, Department of Oral Health Sciences University of Leuven, University Hospitals Leuven Belgium; ^3^ Department of Dental Medicine Karolinska Institutet Sweden; ^4^ Department of Oral and Maxillofacial Surgery University Hospitals Leuven Belgium

**Keywords:** aesthetic, canine, cuspid, impacted, maxillary

## Abstract

Aesthetic appraisal is rarely included in the objective assessment of outcome studies of impacted maxillary canines treatment. The present study aimed to validate a new index for assessing the aesthetic appearance of maxillary canines and adjacent soft tissues. The Department of Oral and Maxillofacial Surgery at University Hospitals Leuven. Four oral‐maxillofacial surgeons, two orthodontists, two prosthodontists, and two lay persons rated 11 maxillary canines and adjacent soft tissues according to the new index. Each of the examiners repeated the examination three times with a 2‐week interval. Twelve relevant aesthetic variables were selected on the basis of the anatomic form, color, and surface characteristics of the canine crown and on the basis of the anatomic form, color, and surface characteristics of the adjacent soft tissues. Intraclass correlation (ICC) coefficient and Fleiss' kappa statistics were performed to analyze the intrarater and interrater agreement. The index proofed to be a reliable assessment tool. Considering the cumulative assessment of the Maxillary Canine Aesthetic Index (MCAI), the mean ICC value for the interrater agreement of the 10 examiners was 0.71, representing a good agreement. Intrarater agreement ranged from 0.10 to 0.91. Interrater agreement (Fleiss' kappa statistics) calculated for each variable ranged from 0.08 to 0.98. The MCAI is a tool in rating aesthetic outcome of impacted canine treatment and adjacent soft tissues. The MCAI can be used to evaluate the aesthetic outcome after surgical exposure or transalveolar transplantation of maxillary canines.

## 1 INTRODUCTION

Aesthetic appraisal is crucial yet rarely included in the objective assessment of outcome studies of impacted maxillary canines treatment. In 2005, Furhauser et al. proposed an excellent index termed the pink aesthetic score (PES), focusing essentially on the soft tissue aspects of an anterior implant restoration. This PES is based on seven variables: mesial papilla, distal papilla, soft‐tissue level, soft‐tissue contour, alveolar process deficiency, soft‐tissue color, and texture. Belser et al. developed an implant restoration index (white aesthetic score) in analyzing a single‐tooth implant. The suitability of the PES/white aesthetic score index for the objective outcome assessment of the aesthetic dimension of anterior single‐tooth implants was confirmed (Belser, Grutter, Vailati, Bornstein, & Weber, 2009; Buser et al., 2008; Furhauser et al., 2005).

Few studies have investigated the aesthetic outcome of previously impacted canines after treatment (Grisar et al., 2018; Parkin, Freeman, Deery, & Benson, 2015; Sampaziotis, Tsolakis, Bitsanis, & Tsolakis, 2017). In the few studies that have been conducted, no clinically detectable difference in tooth color between the exposed teeth and the control groups have been reported (Blair, Hobson, & Leggat, 1998; D'Amico, Bjerklin, Kurol, & Falahat, 2003). Furthermore, shape and position did also not show any differences, yet inclinication was reported to be significantly different in the impacted canine group: 80% of the normally erupted canines had a normal inclination, whereas only 57% of the previously impacted canines had a normal inclination after treatment (D'Amico et al., 2003). Other authors reported that the previously impacted canines were more intruded after treatment (Ling, Ho, Kravchuk, & Olive, 2007; Woloshyn, Artun, Kennedy, & Joondeph, 1994). The three most common reasons given for identifying the previously impacted canines are torque, gingiva, and alignment (Schmidt & Kokich, 2007).

As there are hardly any clinical yet objective assessment methods available, the overall aim of the present study was to introduce the Maxillary Canine Aesthetic Index (MCAI) as a brief, simple, and easy‐to‐use questionnaire to objectively score the aesthetic appearance of maxillary canines. This MCAI is adapted from a combined set of parameters as measured with the highly standardized international pink and white aesthetic scoring system. This study describes the use of the MCAI, meanwhile validating it for assessing the aesthetic appearance of maxillary canines and adjacent soft tissues. As a subobjective, the differential use of the index by different specialists and dental professionals was studied.

## 2 MATERIAL AND METHODS

This retrospective cross‐sectional study was conducted at the Department of Oral and Maxillofacial Surgery, University Hospital Leuven, Belgium. The study protocol was approved by the Ethics Committee of the University Hospitals Leuven, Belgium (s53225).

Out of the literature, 12 variables were selected, which have an influence on the aesthetic result (Table 1). The variables were based on the anatomic form, color, and surface characteristics of the crown and on the anatomic form, color, and surface characteristics of the adjacent soft tissues. All variables and their assessment are described in Tables 1 and 2.

**Table 1:** Maxillary Canine Aesthetic Index variables

	Variables	Explanation	Judgment instructions	Outcome	Figures
Parameters investigating the previously impacted canine	Mesial papilla Distal papilla	Interdental papilla must be in natural position Interdental papilla must be in natural position	Judgment should be made on a 3‐point rating scale	• Complete • Incomplete • Absent	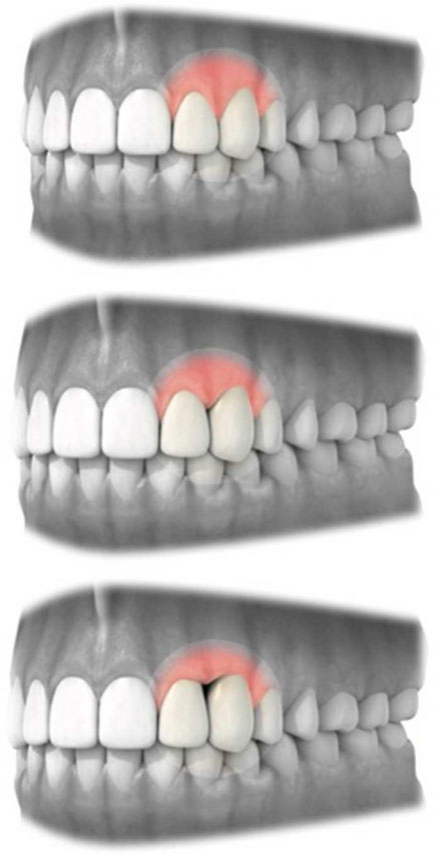
	Marginal gingiva Marginal gingival thickness	Length of the marginal gingiva must be in harmony with the contralateral tooth Thickness of the marginal gingiva must be in harmony with the contralateral tooth	Judgment should be made on a 3‐point rating scale Judgment should be made on a 2‐point rating scale	• Absent, incomplete (<3 mm) or complete (>3 mm) • Thin • Thick	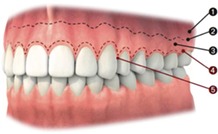 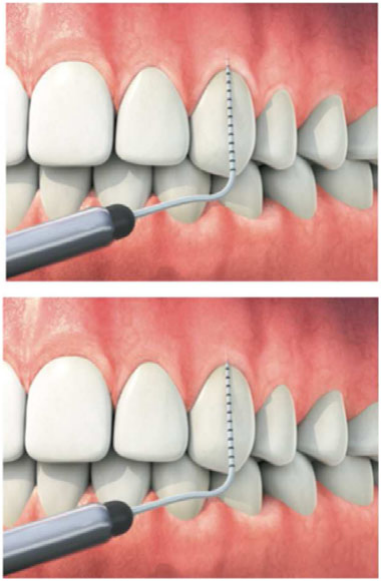
	Recession	Level of displacement of the marginal tissue apical compared with the cemento‐enamel junction (CEJ)	Judgment should be made on a 3‐point rating scale	• No recession • Recession that does not extend to the mucogingival junction (MGJ) • Recession that extends to or beyond the mucovingival junction (MGJ)	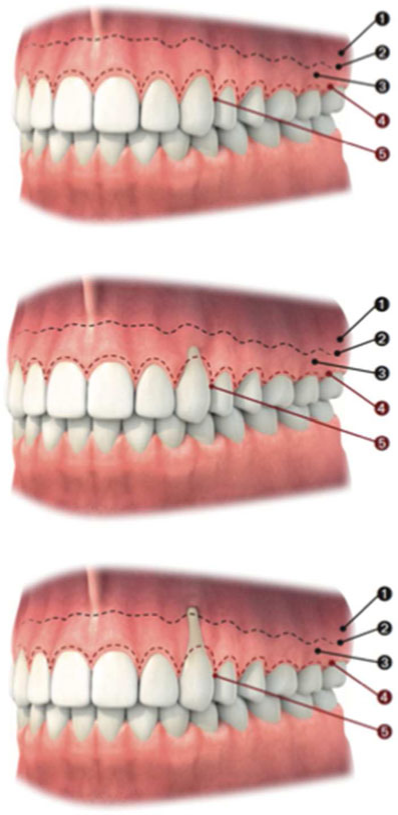
	Mesiodistal crown angulation	Position must be in harmony with the adjacent and contralateral tooth	Judgment should be made on a 3‐point rating scale	• Mesial • Straight • Distal	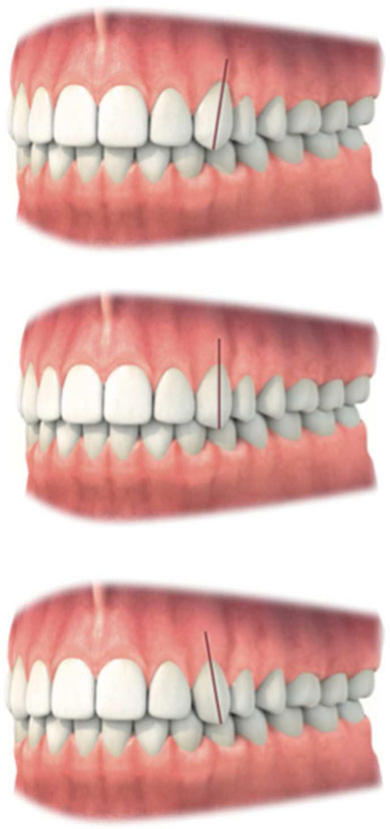
Parameters investigating comparison between both canines	Curvature of marginal gingiva	Curvature of the marginal gingiva must be in harmony with the contralateral tooth	Judgment should be made on a 3‐point rating scale	• Major discrepancy, minor discrepancy or no discrepancy	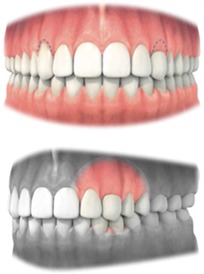
	Soft tissue color and texture Root convexity Tooth morphology	Color (redness) and texture must be in harmony with the contralateral canine and must have a natural appearance Root convexity and its projection through the overlying mucosa must be in harmony with the contralateral canine Tooth morphology must be in harmony with the contralateral canine			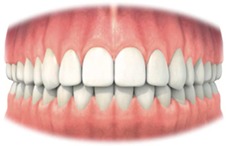 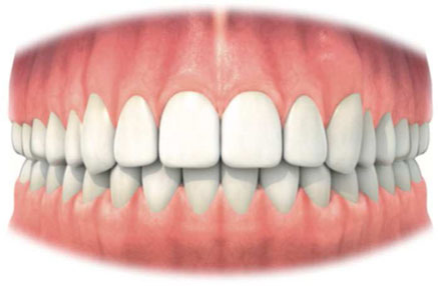 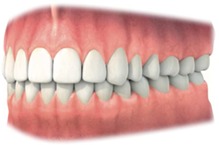
	Vertical tooth position	Vertical position must be in harmony with the adjacent teeth and contralateral canine			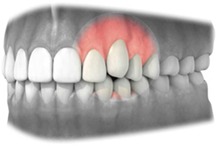
Parameters investigating relation previously impacted canine and neighboring teeth	Buccolingual angulation crown	Buccolingual angulation of the crown must be in harmony with the contralateral canine	Judgment should be made on a 3‐point rating scale	• Major discrepancy • Minor discrepancy • No discrepancy	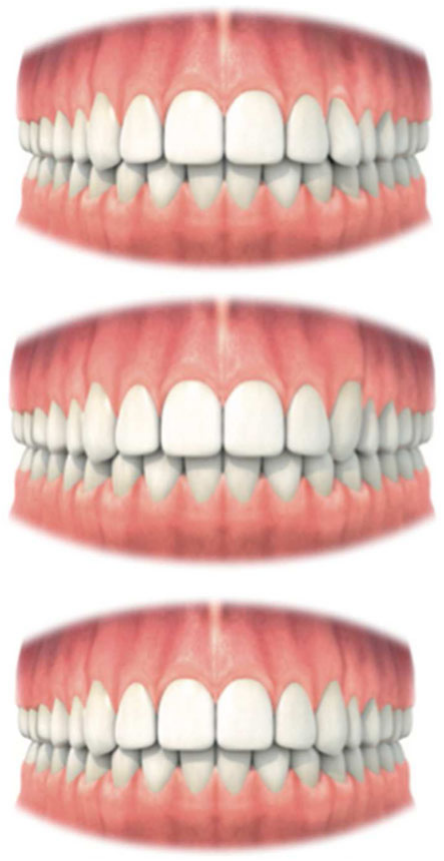

*Note*. 1: Alveolar mucosa; 2: Mucogingival junction; 3: Attached gingiva; 4: Free gingival groove; 5: Free gingiva.

**Table 2:** Maxillary Canine Aesthetic Index scoring sheet

Parameters investigating the previously impacted canine			
	Absent	Incomplete	Complete
Mesial papilla	5	1	0
Distal papilla	5	1	0
Marginal gingiva	5	1 (<3 mm)	0(>3 mm)
Recession	(Apical to MGJ)	(Coronal to MGJ)	(No recession)
	5	1	0
Marginal gingival thickness	Thin	____	Thick
	1	______	0
Mesiodistal crown angulation	Distal	Straight	Mesial
	2	1	0

Parameters investigating comparison between both canines			
	Major discrepancy	Minor discrepancy	No discrepancy
Curvature of marginal gingiva	2	1	0
Soft tissue color and texture	2	1	0
Root convexity	2	1	0
Tooth morphology	2	1	0
Vertical tooth position	2	1	0

Parameters investigating relation previously impacted canine and neighboring teeth			
Buccolingual angulation crown acc. neighboring teeth	2	1	0
Total score	0–3 points = excellent		
	4–8 points = good		
	9–13 points = moderate		
	14 or more points = poor aesthetics		

Rather than using rules for shape and position of the teeth, adjacent and contralateral teeth were used as a reference for normality instead. This allowed maintaining the patient's proportions between the general shape of the face, size, sex, and other teeth. It should be recognized that patients who had treatment for bilateral impacted maxillary canines are more difficult to assess with the MCAI.

In general, MCAI works with a subjective rating scale, according to the following classification: zero points for the desired situations, one point for moderate result, and two or five points for a gross deviation. For the gross deviations, five points are assigned for the variables considered to be the most important for the aesthetic outcome, whereas two points are assigned when the variable is considered to be less important. The higher the score, the worse the aesthetic appearance. It can be noticed that one gross deviation (five points) can never be accepted as to be an excellent outcome.

For the observational tasks, the 10 observers (four oral‐maxillofacial surgeons, two orthodontists, two prosthodontists, and two lay persons) were asked to subjectively score each case with *excellent*, *good*, *acceptable*, and *poor* final outcome. These scorings were correlated with the total objective scores. Initial training and calibration of all observers were performed. Observers were not informed that the patients had been treated for displaced maxillary canine and thus were unaware which were the treated and untreated canine teeth. Observations were performed at T0 (baseline), T1 (2 weeks after T0), and T2 (4 weeks after T0) after randomization. Each of the maxillary canines and the adjacent soft tissues were rated on a form with 12 variables of the rating index. Although blinded for patient history and treatment, observers had to score the canines on their gingival aspects and aesthetics. Intraclass correlation (as described in the original article from Shrout and Fleiss) and Fleiss's kappa tests were calculated to express the intraobserver and interobserver agreement. *P* values in Tables 3 and 4 are the result of a nonparametric bootstrap procedure. The null hypothesis was that there was no difference between each of the combinations of observer types.

**Table 3:** Interrater agreement on final endscore

Observer type	Intraclass correlation
Oral‐maxillofacial surgeons	0.65
Prosthodontists	0.76
orthodontists	0.91
Layman	0.52
Comparison	*P* value
Oral‐maxillofacial surgeons—Prosthodontists	0.50
Oral‐maxillofacial surgeons—Orthodontists	0.05
Oral‐maxillofacial surgeons—Layman	0.47
Prosthodontists—Orthodontists	0.33
Prosthodontists—Layman	0.23
Orthodontisten—Layman	0.02

**Table 4:** Intrarater agreement on final endscore

Observer type	Intraclass correlation
Oral‐maxillofacial surgeons	0.81
Prosthodontists	0.89
orthodontists	0.80
Layman	0.67
Comparison	*P* value
Oral‐maxillofacial surgeons—Prosthodontists	0.50
Oral‐maxillofacial surgeons—Orthodontists	0.97
Oral‐maxillofacial surgeons—Layman	0.42
Prosthodontists—Orthodontists	0.66
Prosthodontists—Layman	0.21
Orthodontisten—Layman	0.60

Observations were carried out in standardized circumstances with dimmed light, on a projection screen with all observers at an equal distance from the screen.

To test reliability of the newly developed index, intraobserver and interobserver agreement must be calculated (Landis & Koch, 1977). Eleven patients (six male, five female; mean age 21.8 years) were randomly selected out of the patients database of the Department of Oral and Maxillofacial Surgery and the Department of Orthodontics, University Hospitals Leuven. Mean follow‐up time was 3.4 years. Six patients had a history of autotransplantation of one maxillary canine, and five patients had a history of surgical exposure of one maxillary canine. All surgical interventions were performed at the Department of Oral and Maxillofacial Surgery, University Hospitals Leuven. All patients had finished their treatment at the final examination. Intraoral images were collected and standardized.

## 3 RESULTS

The interrater and intrarater agreements and comparison between the different groups are listed in Table 3. It can be noticed that especially orthodontists have an excellent interrater reliability. Best intrarater agreement was noticed within the group of prosthodontists.

Interrater agreement (Fleiss' kappa statistics) ranged from 0.52 (layman) to 0.91 (orthodontists). Lowest scores were noted within the layman group assessing marginal thickness of the gingiva and mesial papilla. Highest scores were noted within the group of orthodontists and maxillofacial surgeons assessing gingival recession.

**Figure 1:**
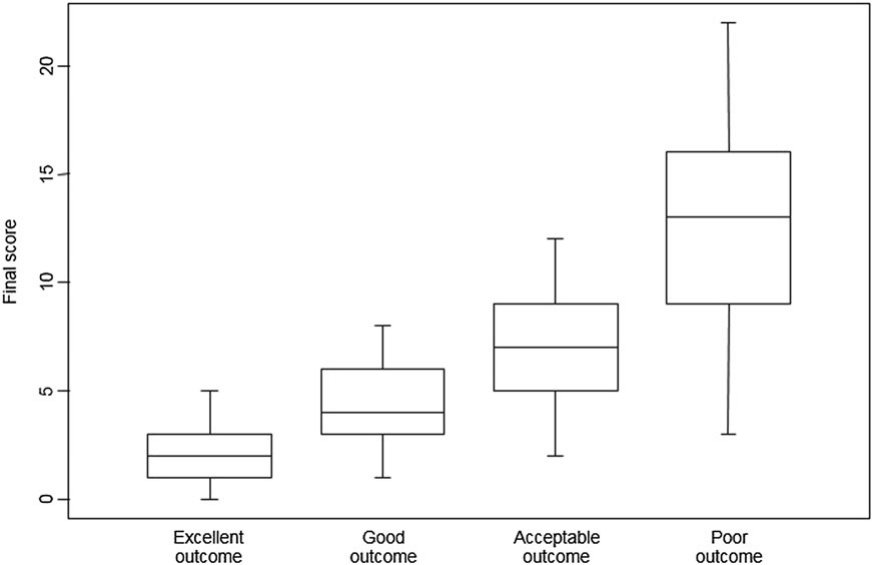
Box plots displaying correlations objective and subjective scoring. X‐axis represents the subjective scoring as given by the different observers. Y‐axis represents the corresponding mean final objective score on the Maxillary Canine Aesthetic Index. Cut‐off values for correlation of objective and subjective scoring were obtained

The subjective scoring of each observer was correlated with the total scores (Figure 1). A discriminant validity testing was performed by comparing the final score between the subjective scoring classes. A residual analysis showed that data were normally distributed around their mean, although some heteroscedasticity was observed. For this reason, we applied weighted ANOVA and applied a correction for simultaneous hypothesis testing according to Tukey. It appears that there is a significant discrimination between the groups.

Based upon these results, the following classification was proposed (Table 5). A total objective score of 0–3 points correlated with an excellent final outcome, a total objective score of 4–8 points with a good final outcome, a total objective score of 9–13 points with an acceptable final outcome, and a total objective score of 14 or more points with a poor final outcome. Receiver‐operating characteristic curve analysis was performed for testing of index sensitivity (ranging from 82% to 100%).

**Table 5:** Correlation final score MCAI with outcome

Total score MCAI	Final outcome
0–3	Excellent
4–8	Good
9–13	Acceptable
>13	Poor

*Note*. MCAI: Maxillary Canine Aesthetic Index.

## 4 DISCUSSION

In the present study, we introduced a new index (MCAI) and validated it. It was developed considering the lack of a standardized method of evaluating and measuring aesthetics after treatment of impacted maxillary canines. The goal was to develop an index that could be used in both research and clinical settings as a guideline for diagnosing and documenting aesthetics.

The best interobserver agreement (Table 3) was found between the orthodontists. Intraobserver results concerning the final score (Table 4) indicated an excellent agreement in the three groups of medically trained observers. For the layman group, there is a good agreement.

It has been chosen to use the adjacent and contralateral tooth as a reference and not the generally accepted rules for shape and position of teeth. One should always take into account the harmony with other teeth, even if gross deviations exist with aesthetic principles.

As consistency is a key feature of the aesthetic evaluation, the high intrarater and interrater consistency reliability were considered high‐quality features of the MCAI. The examiner was trained and calibrated in the use of the index before the evaluation sessions, which confirms the need for those steps. This step contributed to the good results. Ratings have been carried out under standardized viewing conditions for all observers. Thus, observation settings are standardized, without interference of the possible opinion of the patient. On the other hand, real color and surface characteristics were more difficult to examine. Also, a clinical chairside evaluation would contribute to a better comparison with the contralateral tooth.

These initial results with the aesthetic index are very promising, but its practical use as a standard procedure has to be confirmed in a large‐scale clinical study. The index could be a very useful tool in scientific research and in a clinical setting. It makes comparison between various surgical procedures possible. The index could also give a better, objective, insight in one's own aesthetic results in daily practice.

## 5 CONCLUSIONS

The current investigation presents the MCAI, an objective tool for rating aesthetics of maxillary canines and adjacent soft tissues after surgical treatment. Clinicians might find it useful in daily clinical practice and scientific research. However, one must be aware that this index only judges the aesthetic and not the functional outcome of the canine. A poor aesthetic result does not imply malfunction.

## CONFLICT OF INTEREST

The authors report no conflicts of interest related to this study.
